# Interference-Enhanced Absorption in Miniaturized Graphene Plasmonic Terahertz Detectors via Substrate-Defined Fabry−Pérot Cavities

**DOI:** 10.3390/nano16130794

**Published:** 2026-06-26

**Authors:** Runli Li, Shaojing Liu, Ximiao Wang, Hongjia Zhu, Yongsheng Zhu, Shangdong Li, Huanjun Chen, Shaozhi Deng

**Affiliations:** 1 State Key Laboratory of Optoelectronic Materials and Technologies, School of Electronics and Information Technology, Sun Yat-sen University, Guangzhou 510275, China; lirli3@mail2.sysu.edu.cn (R.L.); liushj83@mail.sysu.edu.cn (S.L.); wangxm45@mail3.sysu.edu.cn (X.W.); zhuhj3@mail2.sysu.edu.cn (H.Z.); zhuysh23@mail2.sysu.edu.cn (Y.Z.); lishd8@mail2.sysu.edu.cn (S.L.); stsdsz@mail.sysu.edu.cn (S.D.); 2 Guangdong Province Key Laboratory of Display Material and Technology, School of Electronics and Information Technology, Sun Yat-sen University, Guangzhou 510275, China

**Keywords:** graphene terahertz detector, plasmons, interference-enhanced absorption, photothermoelectric effect, Fabry–Pérot cavity, terahertz imaging

## Abstract

Two-dimensional (2D) material terahertz (THz) detectors offer a promising platform for compact, room-temperature detection, yet their performance is fundamentally constrained by weak absorption in atomically thin layers. Here, we demonstrate a graphene plasmon polariton atomic cavity (PPAC) THz detector in which intrinsic graphene plasmon absorption is enhanced through vertical cavity-assisted field redistribution. By incorporating a metallic back reflector beneath a silicon substrate of designed thickness, a Fabry–Pérot (FP) interference cavity is formed that positions the standing-wave antinode near the graphene plasmonic layer. Electromagnetic simulations reveal that the Fabry–Pérot cavity itself primarily redistributes the vertical electromagnetic field, thereby enhancing the local in-plane driving field responsible for intrinsic graphene plasmon excitation. Experimental measurements at the optimized cavity condition confirm a pronounced increase in plasmon-induced photothermoelectric response, consistent with the predicted absorption enhancement. As a result, the detector exhibits an approximately 30-fold increase in responsivity compared with the corresponding structure without the cavity, while maintaining a fast response time below 130 μs. The detector further enables discrimination of concealed polar and nonpolar liquids through continuous-wave THz imaging at 2.52 THz, achieving a discrimination speed 30-fold faster than that of conventional time-domain spectroscopy. This result highlights the potential of cavity-enhanced intrinsic plasmon absorption for compact, high-sensitivity, and high-speed THz photodetection.

## 1. Introduction

To meet the growing demands of high-speed communication, real-time imaging, and on-chip system integration, terahertz (THz) photodetectors must simultaneously achieve room-temperature operation, high sensitivity, fast response, and device miniaturization [1,2,3,4,5,6,7]. However, conventional THz detection technologies remain constrained by fundamental trade-offs among these performance metrics. Thermal detectors, such as pyroelectric sensors and Golay cells, operate at room temperature but suffer from inherently slow response times [8,9,10]. Electronic devices, including Schottky diodes and high-electron mobility transistors, offer faster operation yet are typically limited in bandwidth and sensitivity [11,12]. In contrast, detectors based on quantum wells and superconductors exhibit superior sensitivity and noise performance but require cryogenic cooling, which hinders scalable integration [13,14,15]. Consequently, achieving high sensitivity and high-speed detection in compact, room-temperature THz detectors remains a central challenge, limiting the practical deployment and large-scale integration of THz systems. These limitations underscore the need for alternative physical mechanisms and material platforms capable of reconciling strong light–matter interaction with scalable device integration. In this context, 2D materials have emerged as a promising platform, offering ultrathin active layers, high carrier mobility, and compatibility with on-chip integration, while supporting electrically tunable electromagnetic resonances that enable strong light–matter interaction in the THz regime [16,17,18]. In parallel, multifunctional thin-film materials such as multiferroic BiFeO_3_-based systems have also attracted attention for integrated sensing and microelectronic applications owing to their room-temperature operability and scalable fabrication compatibility [19,20,21]. However, achieving strong THz light–matter interaction in compact room-temperature platforms remains challenging.

In recent years, graphene has attracted significant interest for THz micro/nano photodetection owing to its high carrier mobility, electrically tunable band structure, and efficient photoelectric conversion mechanisms [22,23,24,25,26]. Notably, patterning graphene into micro- and nanostructures enables the excitation of localized graphene plasmons, which concentrate electromagnetic energy at subwavelength scales and substantially enhance THz absorption compared with continuous films [27,28]. For example, graphene plasmon polariton atomic cavity (PPAC) detectors employing engineered microdisk arrays have demonstrated multi-parameter THz detection of polarization, wavelength, and amplitude while maintaining a compact footprint without external antenna focusing [29]. Despite these advantages, the responsivity of graphene-based THz detectors remains fundamentally limited by the atomic thickness of graphene. Even under plasmonic confinement, the overall absorption cross-section of a single-layer material is intrinsically small, which constrains photocarrier generation and thus device sensitivity. Consequently, whether based on unpatterned graphene films directly illuminated by free-space radiation or on plasmonic micro/nanostructures, insufficient absorption remains a primary bottleneck for achieving high-responsivity, room-temperature THz detection.

To overcome this limitation, various field-enhancement strategies have been explored. Integration with metallic antennas, metamaterial absorbers, or metasurfaces can enhance field localization and increase absorption through hybrid plasmon–antenna or plasmon–cavity mode interactions [17,18,22,23]. In particular, substrate-based FP interference combined with metallic back reflectors has been employed to enhance graphene-based THz detectors integrated with antennas or metamaterial resonators [30,31,32,33,34]. In these architectures, the cavity primarily serves to reinforce the externally engineered resonant structures that couple free-space radiation into graphene. While effective, these approaches typically rely on multilayer stacking, patterned metallic resonators, or large-area focusing structures, which increase fabrication complexity and limit scalability and CMOS compatibility. A more streamlined strategy would be to directly couple a substrate FP cavity to the intrinsic plasmonic resonance of patterned graphene nanostructures, thereby enhancing absorption without auxiliary antennas or metamaterials. In such a scheme, the FP cavity itself acts primarily as a lossless electromagnetic resonator that redistributes the vertical standing-wave field distribution, allowing the out-of-plane electric-field antinode to be aligned with the graphene layer. Under resonant conditions, the enhanced local in-plane driving field strengthens the excitation amplitude of intrinsic graphene plasmons, thereby increasing dissipative absorption within the graphene channel without introducing auxiliary antenna or metamaterial resonators. Under resonant conditions, constructive interference increases the local electric-field amplitude at the graphene plane, thereby strengthening the in-plane carrier oscillations responsible for plasmon excitation. As a result, the excitation strength of graphene plasmons is governed not only by lateral geometric confinement but also by the cavity-imposed vertical boundary conditions. When the FP resonance spectrally aligns with the intrinsic graphene plasmon mode, enhanced light–plasmon coupling can be achieved, leading to increased THz absorption within the graphene channel. However, despite its clear advantages, such direct coupling between a substrate FP cavity and intrinsic graphene plasmon modes has not yet been explored.

Here, we report a graphene THz photodetector architecture that enhances light–matter interaction through cavity-controlled out-of-plane electromagnetic confinement, without relying on antenna- or metamaterial-assisted coupling. The device integrates a graphene PPAC array with an interferometric enhancement of absorption (IEA) structure, where a metallic back reflector and the substrate form an FP cavity that coherently reshapes the vertical electric-field distribution. By optimizing the spacing between the reflector and the patterned graphene layer, the standing-wave antinode can be positioned at the graphene plane, enabling constructive interference and pronounced local field enhancement. The intensified out-of-plane field strengthens coupling to the intrinsic graphene plasmon resonance modes, increasing plasmon excitation efficiency and amplifying the in-plane carrier heating and temperature gradient. This process results in an enhanced plasmon-driven photothermoelectric (PTE) response. Systematic numerical simulations, together with experimental measurements, reveal the dependence of PTE responsivity on cavity spacing and excitation frequency, confirming the role of vertical mode positioning in boosting absorption. Experimentally, the optimized cavity condition delivers a 30-fold enhancement in PTE responsivity compared to a non-cavity reference device, while preserving a fast response time below 130 μs. We further demonstrate continuous-wave THz imaging at 2.52 THz capable of distinguishing optically transparent liquids. The discrimination speed is 30-fold faster than that of conventional pulsed time-domain spectroscopy, underscoring the advantages of this cavity-engineered architecture for compact, high-responsivity, room-temperature, and high-speed THz sensing and imaging applications.

## 2. Method

Sample Preparation. A large-area (>1 × 1 cm^2^) monolayer graphene film (Jiangsu XFNANO Materials Tech. Co., Ltd., Nanjing, China) was first transferred onto the oxidized side of a double-side polished silicon substrate with a resistivity over 20,000 Ω·cm (Nanjing MKNANO Tech. Co., Ltd., Nanjing, China) via a wet transfer method. Commercial CVD-grown graphene was selected to improve device-to-device uniformity and fabrication scalability compared with mechanically exfoliated graphene. During the transfer process, the substrate cleaning and transfer conditions were optimized to minimize wrinkles, cracks, and polymer residues. The graphene was then patterned using maskless lithography (UV Litho-ACA, Tuotuo Technology, Suzhou, China) followed by oxygen plasma etching. Particular attention was paid to the plasma etching conditions to ensure a uniform pattern was generated while avoiding excessive graphene damage. Subsequently, another round of maskless lithography was performed, and electrodes were fabricated by thermal evaporation of 10 nm Cr and 70 nm Au (ZhongNuo Advanced Material (Beijing) Technology Co., Ltd., Beijing, China). The electrode-coated surface was spin-coated with photoresist for protection, while selected regions on the back side of the substrate were covered and deposited with 10 nm Cr and 100 nm Au via evaporation. Finally, the device was completed through a lift-off process. Raman spectroscopy shows that the graphene in the channel has good monolayer properties and high etching quality (Figure S7, Supporting Information). The morphologies of the fabricated detectors were characterized using scanning electron microscope (SEM).

Measurement. A continuous-wave terahertz laser (FIRL 100, Edinburgh Instruments Ltd., Livingston, UK) operating at 2.52 THz was used for device response and photocurrent dual-focus scanning imaging. The beam, with a spot diameter of 1 mm, was modulated into a quasi-square wave using an optical chopper before illuminating the device. To investigate the trend of device response with frequency, a microwave signal generator (SMB100A, Rohde & Schwarz GmbH & Co. KG, Munich, Germany) provides the fundamental frequency to the electronic source (SGX, Virginia Diodes, Inc., Charlottesville, VA, USA) paired with an external three-times multiplier whose output power curve is shown in the Figure S8, Supporting Information. The signal from the device electrodes was transmitted via coaxial cable to a low noise transimpedance amplifier (DLPCA-200, FEMTO Messtechnik GmbH, Berlin, Germany). The amplified voltage signal was then processed by a lock-in amplifier (OE1201, Sine Scientific Instrument, Guangzhou, China) to extract the photoresponse by noise suppression. By observing the cross-section using SEM, we measured the thickness of the silicon substrate (Figure S9, Supporting Information).

Responsivity Calculation. The voltage signal $V_{\mathrm{lock}}$ measured by the lock-in amplifier is related to the photocurrent $I_{\mathrm{ph}}$ by [35],

(1) $$I_{\mathrm{ph}}=\frac{2\pi \sqrt{2}V_{\mathrm{lock}}}{4G}$$

where G is the gain of the preamplifier. Since no bias voltage was applied, the voltage responsivity $R_{v}$ (in mV/W) is defined as:

(2) $$R_{v}=\frac{I_{\mathrm{ph}}\times R}{P_{\mathrm{eff}}}$$

(3) $$P_{\mathrm{eff}}=\frac{S_{D}}{S_{B}}\times P_{B}$$

Here, $I_{\mathrm{ph}}$ is the photocurrent, R is the resistance of the detector, $P_{\mathrm{eff}}$ is the effective optical power, $S_{D}$ is the area of the device channel, $S_{B}$ is the beam spot area, and $P_{B}$ is the power of the terahertz beam. In the present measurements, the preamplifier exhibits a much lower input impedance than the detector, and the readout configuration is designed to maximize current extraction efficiency rather than achieve conventional power matching. These considerations suggest that the detector is well suited for rapid THz imaging applications.

NEP calculation [36]:

(4) $$NEP=\frac{i_{\mathrm{noise}}}{R_{v}}\sqrt{B}$$

where $i_{\mathrm{noise}}$ is the total noise current and B is the bandwidth. The noise power density spectrum of the device was measured using a low-frequency noise testing system (LFN-2000, XINJIAN Semilab, Wuxi, China).

Simulations. Finite-difference time-domain (FDTD) simulations were performed using Lumerical FDTD Solutions 2020 R2.4 (Ansys Lumerical, Vancouver, BC, Canada) to investigate the optical response of the proposed structure. The geometric model was constructed based on the design dimensions, with the refractive indices of the silicon and silicon dioxide layers set to 3.4 and 1.97, respectively. The refractive-index dispersion of high-resistivity silicon over the investigated THz range is relatively weak [37,38]. The fluctuation of the refractive index across 0.5 to 7.7 THz is less than 0.1%. We therefore used the standard constant-index approximation in the simulations. Owing to the atomic-scale thickness of monolayer graphene, which is several orders of magnitude smaller than the THz wavelength, graphene was modeled as a two-dimensional surface-conductivity layer rather than a volumetric material, following the standard treatment widely adopted in simulating graphene plasmonics and THz electrodynamics studies [39,40,41]. The chemical potential and scattering rate were set to 0.3 eV and 0.003 eV, respectively, while other parameters remained at their default values. Perfect electrical conductors (PECs) were used to replace the metals in the modeling.

Symmetric and antisymmetric boundary conditions are used within the FDTD domain to shorten computation time, while a perfectly matched layer (PML) with a thickness greater than half the maximum wavelength is used outside the FDTD domain. The structure is illuminated using a broadband electromagnetic source, and the spectral response within the target wavelength range is calculated. The actual simulation time is automatically determined by the auto shutoff level to ensure that the electromagnetic field is sufficiently attenuated before termination.

Numerical simulations were also performed using the finite element solver implemented in COMSOL Multiphysics 6.2 (COMSOL AB, Stockholm, Sweden). A three-dimensional model was constructed to investigate the electromagnetic and thermoelectric response of the graphene-based detector. The simulated device consists of a graphene layer placed on a dielectric substrate composed of SiO_2_ and Si. Periodic patterned graphene structures were introduced in the channel region. Two metal electrodes were placed on the left and right sides of the graphene channel. The electrode width was 5 μm and the metal thickness was 80 nm. The optical response of graphene was described using a Drude-type conductivity model derived from the Kubo formalism. The surface conductivity of graphene is expressed as [42],

(5) $$\sigma \left(\omega \right)=-\frac{e^{2}}{\pi \hbar^{2}}\frac{iE_{F}}{\omega -i/\tau }$$

where *E_F_* is the Fermi energy and *τ* is the carrier relaxation time. In the simulations, the Fermi energy was set to −0.3 eV and the relaxation time was 1 × 10^−13^ s.

A plane wave excitation was used as the incident electromagnetic source. Perfectly matched layers (PMLs) were implemented around the simulation domain to suppress artificial reflections.

The absorbed optical power in graphene was converted into a heat source to evaluate the photothermal response of the device. Heat transport in the graphene layer and substrate was modeled using the heat conduction equation. The photothermoelectric voltage generated in the graphene channel was calculated based on the spatial distribution of the Seebeck coefficient and the temperature gradient. The spatial distribution of Seebeck coefficients in the channel is calculated using Equation (6) [43],

(6) $$S=-\frac{\pi^{2}k_{B}^{2}T}{3\left|e\right|}\frac{1}{\sigma }{\left.\frac{d\sigma }{dE}\right|}_{E=E_{F}}$$

where σ is the electrical conductivity of graphene.

The Seebeck coefficient of graphene was modeled as a function of gate voltage and carrier mobility using analytical expressions implemented in the simulation model.

## 3. Results and Discussion

**Figure 1:**
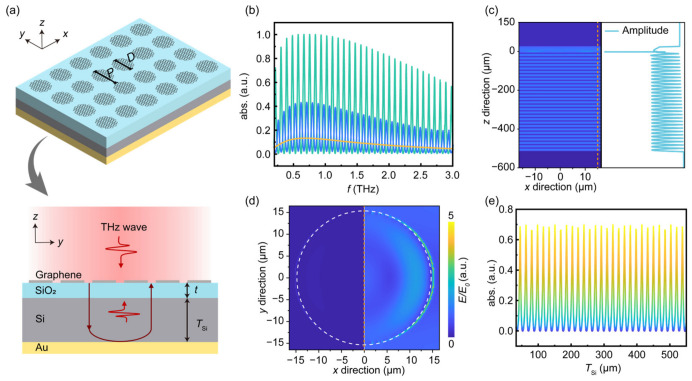
Principle of interference-enhanced absorption in a graphene plasmon polariton atomic cavity (PPAC) array. (**a**) Schematic illustration of the graphene PPAC array (top view) and the corresponding substrate configurations (cross-sectional side view). The deep red arrow indicates the propagation direction of terahertz waves within the substrate. (**b**) Simulated THz absorption spectra for three configurations: semi-infinite substrate (orange), finite-thickness silicon substrate (blue), and IEA structure with metallic back reflector (green). (**c**) Simulated out-of-plane electric-field distribution (*x*–*z* plane) for the IEA structure, showing vertical standing-wave formation. (**d**) In-plane electric-field distribution within the graphene layer without (left) and with (right) IEA enhancement. (**e**) Calculated absorption intensity as a function of substrate thickness at 2.52 THz.

To maximize device absorption, we investigate the THz response of a graphene PPAC array composed of microdisks (diameter *D* = 30 μm, period *P* = 33 μm) under three substrate configurations using finite-difference time-domain (FDTD) simulations (Figure 1a). For an effectively semi-infinite silicon substrate with a 300 nm SiO_2_ layer, the absorption spectrum exhibits a single broad resonance peak (Figure 1b, orange curve), corresponding to the intrinsic plasmonic mode of the patterned graphene array. When the substrate is replaced by a finite-thickness (500 μm) high-resistivity silicon wafer, the absorption spectrum evolves into an FP interference pattern (Figure 1b, blue curve). Multiple absorption maxima emerge due to interference between the incident THz wave and the wave reflected from the backside silicon–air interface. In this configuration, the vertical electromagnetic field distribution becomes modulated by the substrate thickness, leading to periodic spectral variation superimposed on the intrinsic plasmon resonance. Introducing a perfect electric conductor (PEC) beneath the substrate further modifies this interference process. The metallic back reflector enforces coherent superposition of the incident and reflected waves, transforming the structure into an IEA cavity. Under this condition, the FP resonances become sharper and more pronounced, and the peak absorption is approximately doubled compared to the non-reflector case (Figure 1b, green curve). Importantly, the relatively narrow FP interference envelope is modulated by the broader intrinsic plasmon resonance of the PPAC array, indicating that the FP cavity primarily redistributes the vertical electromagnetic field, while the dissipative absorption process itself remains governed by intrinsic graphene plasmon excitation.

This absorption-enhancement mechanism is conceptually distinct from previously reported FP-assisted graphene detectors based on antenna- or metamaterial-mediated coupling. In those systems, the FP cavity primarily reinforces the resonance of metallic antennas or metamaterials, which subsequently couple energy into graphene. In contrast, the present IEA structure can be treated as a lossless FP resonator that directly modulates the vertical field distribution experienced by the graphene plasmonic array. As illustrated in Figure 1a (bottom panel), the absorption maxima follow the dual-beam interference condition,

(7) $$\frac{\lambda }{2}+\left(T_{\mathrm{Si}}\times n_{\mathrm{Si}}+T_{{\mathrm{SiO}}_{2}}\times n_{{\mathrm{SiO}}_{2}}\right)\times 2=m\times \lambda ,\;m=1,2,3\ldots$$

where $T_{\mathrm{Si}}$, $T_{{\mathrm{SiO}}_{2}}$, $n_{\mathrm{Si}}$, and $n_{{\mathrm{SiO}}_{2}}$ denote the thicknesses and refractive indices of the silicon substrate and oxide layer, respectively. For $T_{\mathrm{Si}}$ = 500 μm and $T_{{\mathrm{SiO}}_{2}}$ = 300 nm, Equation (7) predicts a uniform resonance spacing of $\Delta T_{\mathrm{si}}=\frac{\lambda }{2n_{\mathrm{si}}}$, in good agreement with the FDTD results (Figure 1b and Figure S1, Supporting Information). These resonances are determined solely by the cavity optical length and therefore enable deterministic control of the out-of-plane standing-wave distribution without substantially altering the intrinsic plasmon resonance frequency defined by the graphene geometry. For *x*-polarized THz illumination, tuning $T_{\mathrm{Si}}$ shifts the vertical standing-wave pattern such that an electric-field antinode can be positioned precisely at the graphene plane (Figure 1c). This vertical mode positioning enhances the local electric-field amplitude experienced by the graphene PPACs, thereby strengthening in-plane plasmon polariton oscillations compared with the configuration without the back reflector (Figure 1d). The enhanced plasmon excitation increases dissipative absorption within the graphene channel, leading to amplified overall absorption of the PPAC array. Importantly, for a fixed intrinsic plasmon resonance frequency, the absorption maximum of the IEA structure can be spectrally tuned by adjusting $T_{\mathrm{Si}}$, resulting in uniformly spaced resonant peaks (Figure 1e). This tunability enables precise alignment of a cavity resonance with a targeted plasmonic mode, allowing the operating frequency of the detector to be engineered through cavity design rather than additional antenna or metamaterial structures. Notably, the cavity modifies the excitation amplitude of the plasmonic modes while leaving their resonance frequency primarily determined by the microdisk geometry.

**Figure 2:**
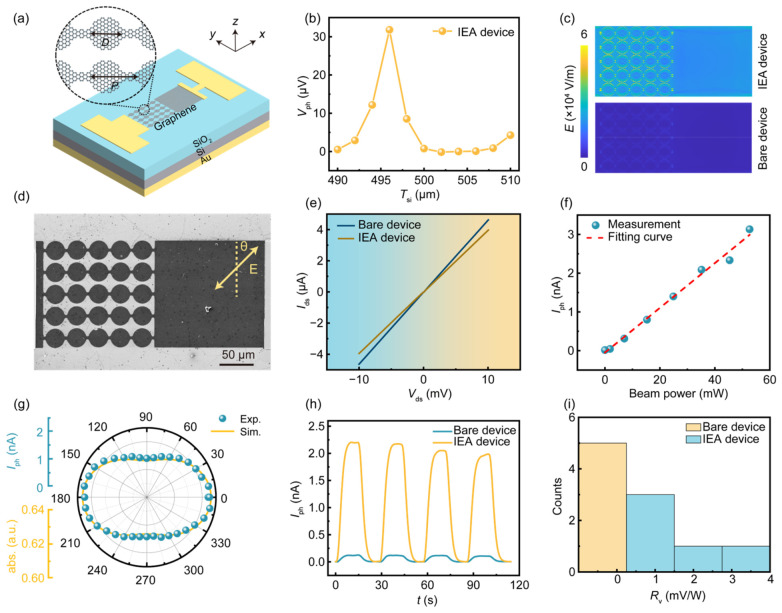
Experimental characterizations of the IEA graphene PPAC THz detector. (**a**) Schematic illustration of the graphene PPAC array integrated with the IEA structure. The device consists of a graphene microdisk PPAC array patterned on a Si/SiO_2_ substrate with a metallic back reflector forming an FP cavity. (**b**) Simulated photovoltage response as a function of substrate thickness for the IEA device, demonstrating cavity-modulated PTE response. (**c**) Simulated in-plane electric-field distribution within the graphene channel for devices with and without IEA structure, illustrating cavity-controlled field enhancement at the graphene layer. (**d**) Scanning electron microscopy image of the fabricated graphene PPAC detector. The yellow arrow illustrates the excitation polarization. (**e**) *I*–*V* characteristics of detectors with and without the IEA structure. (**f**) Power-dependent photoresponse of the IEA device under THz illumination. (**g**) Polarization-dependent photocurrent of the IEA device (green symbols). The yellow line shows the simulation absorption of the device upon different polarization excitations. (**h**) Temporal switching characteristics comparing devices with and without the IEA structure. (**i**) Statistical distribution of responsivity for *N* = 5 independently fabricated devices with and without the IEA structure; the standard deviations are 1.29 mV/W and 0.02 mV/W respectively.

Having established that the IEA structure can control the vertical electromagnetic field distribution and thereby strengthen the excitation of intrinsic graphene plasmons, we next investigate how this cavity-enabled field modulation translates into THz detection performance. To this end, the IEA cavity is integrated with a graphene PPAC detector, forming a device where the vertical FP field distribution governs the excitation of the PPAC plasmonic resonance. Upon THz illumination, the incident electromagnetic field excites localized plasmon polariton modes within the graphene microdisk array. Because the plasmonic absorption is spatially nonuniform across the graphene channel, arising from both the microdisk geometry and the asymmetric device configuration in which one half of the channel contains the PPAC array while the other half remains unpatterned graphene (Figure 2a,d), the resulting plasmon-induced Joule heating is likewise spatially nonuniform. This asymmetric carrier heating establishes an in-plane electron temperature gradient along the graphene channel, which generates a PTE voltage through the Seebeck effect. Full-channel multiphysics simulations (see Section 2 for details) reveal that the photovoltage measured between the two terminals of the device is strongly modulated by the substrate thickness that determines the FP standing-wave condition (Figure 2b). When the cavity resonance aligns with the intrinsic plasmon resonance of the PPAC array, the vertical standing-wave antinode coincides with the graphene layer, maximizing the local electric-field amplitude that drives plasmon oscillations (condition of *T*_Si_ = 496 μm as shown in Figure 2b). Consequently, the local in-plane driving field responsible for plasmon excitation within the graphene channel is significantly enhanced (Figure 2c), resulting in stronger localized THz wave dissipation and carrier heating.

The spatially varying plasmon-induced heating produces a pronounced temperature gradient across the graphene channel, which becomes maximum for an optimized $T_{\mathrm{Si}}$ of 496 μm (Figure S2, Supporting Information). Because the PTE voltage scales with the temperature difference along the channel, this enhanced temperature gradient directly translates into a larger photovoltage. These results demonstrate that the FP cavity-controlled vertical field distribution not only enhances optical absorption but also governs the PTE processes that ultimately determine the detector responsivity. Devices with and without the IEA structure were fabricated using standard microfabrication processes (Figure S3, Supporting Information). The current–voltage (*I*–*V*) characteristics exhibit linear behavior, indicating Ohmic contact between the graphene channel and the electrodes (Figure 2e). The device resistance is approximately 2530 Ω. Importantly, introducing the IEA structure does not alter the resistance distribution of the detector (Figure 2e and Figure S4, Supporting Information); both device types show uniform and stable resistances within the same order of magnitude, indicating that the cavity integration primarily modifies the optical response rather than the electrical transport properties. As the incident THz power increases, the photoresponse exhibits a linear dependence (Figure 2f), yielding a peak responsivity of 3.3 mV/W.

When the polarization direction of the incident THz radiation is varied, the photocurrent shows a moderate polarization dependence. In principle, an isolated graphene microdisk with centrosymmetric geometry is expected to exhibit nearly isotropic THz absorption. The observed anisotropy therefore does not originate from the disk geometry itself, but from symmetry breaking introduced at the device level. Specifically, the observed anisotropy may arise from three extrinsic structural factors: the metal electrode configuration, the graphene interconnect geometry, and the asymmetric current collection pathway across the channel. Numerical simulations show that the metal electrodes do not induce pronounced local field concentration in the graphene channel (Figure S5, Supporting Information), suggesting that their direct contribution is limited. By contrast, the patterned graphene interconnect strips break the rotational symmetry of the microdisk array, so that the relative orientation between the incident THz field and the strips modifies the local field distribution and the coupling efficiency into the plasmonic modes, consistent with the simulated polarization-dependent absorption in Figure 2g (yellow curve). In addition, the asymmetric current collection pathway can further convert this absorption anisotropy into an anisotropic PTE response. Taken together, these results indicate that the anisotropic photoresponse is dominated primarily by symmetry breaking introduced by the graphene interconnect strips, with an additional contribution from asymmetric current collection.

To more clearly compare the performance of detectors with and without the IEA structure, their temporal switching responses under identical illumination conditions are shown in Figure 2h. The IEA device consistently exhibits a larger signal amplitude while preserving stable and repeatable operation. A statistical analysis of the responsivity distributions further highlights this enhancement (Figure 2i). Compared with the bare devices, which show responsivities concentrated near zero with an average value of 0.068 mV/W, the IEA devices display a distinctly shifted distribution toward higher responsivity, yielding an average value of 2.119 mV/W. This corresponds to an enhancement of nearly 30-fold, confirming that the IEA architecture provides a robust and statistically reproducible enhancement of detector responsivity. We note that gate-dependent measurements could further enhance the responsivity by tuning the graphene Fermi level through electrostatic gating. However, the present work is focused on establishing cavity-enhanced intrinsic plasmon absorption under fixed operating conditions. Implementing gate tunability would require substantial redesign of the current device architecture and is therefore left for future investigation.

**Figure 3:**
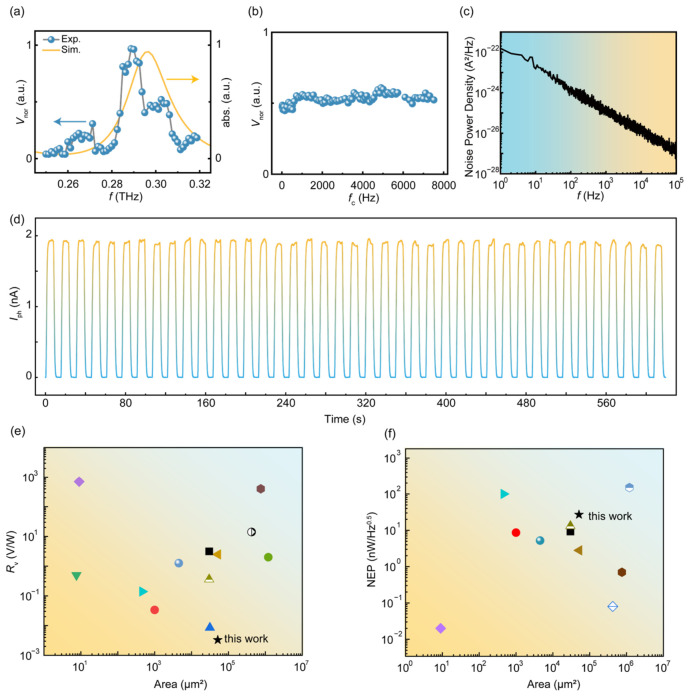
Detection performances of the IEA graphene PPAC THz detector. (**a**) Frequency-dependent responsivity of the IEA graphene THz detector. The normalized device photovoltage response corresponds to the left coordinate axis, and the absorption characteristic curve of the IEA structure corresponds to the right coordinate axis, as indicated by the same-color arrows. (**b**) Photovoltage response as a function of modulation frequency of the THz illumination. (**c**) Noise power spectral density of the device measured at zero bias. (**d**) Long-term operational stability of the detector. (**e**,**f**) Comparison of the responsivity (**e**) and NEP (**f**) of the IEA graphene PPAC THz detector with those of previously reported room-temperature graphene THz detectors, the black five-pointed star represents this work, and other data points are from the Table S1, Supporting Information.

For the IEA-enhanced detector, the responsivity depends not only on the substrate thickness but also on the frequency of the incident THz radiation, because both the excitation efficiency of the graphene plasmon resonance and its spectral overlap with the FP cavity mode determine the absorption enhancement at the graphene layer. Owing to the limited discrete output frequencies available from the gas laser used in our measurements, the frequency dependence was further examined using a frequency-tunable electronic source from Virginia Diodes, Inc. (VDI, see Section 2 for details). We therefore focused on the 0.25–0.32 THz range, which corresponds to one of the characteristic FP resonances of the IEA structure, as illustrated in Figure 1b. The measured responsivity in this frequency window agrees well with the simulated trend (Figure 3a), confirming that the cavity-enhanced absorption is directly translated into an enhanced detector response. The response speed is another key performance metric for THz detectors. Even when the modulation frequency was increased to 7.8 kHz, which is limited by the chopping frequency of the chopper we used, no obvious attenuation of the photoresponse was observed (Figure 3b), indicating that the experimentally measured upper bound of the response time is shorter than 130 μs under the present modulation conditions. Such result suggests that the introduction of the IEA structure will not measurably degrade the intrinsic speed of the detector within the experimentally accessible bandwidth, because the FP cavity only redistributes the incident electromagnetic field and enhances the absorption at the graphene layer, without introducing additional slow electrical or thermal components. The response speed therefore remains governed primarily by the intrinsic PTE dynamics of the graphene PPAC detector itself, which can be up to nanosecond-scale as shown in our previous study [29]. Moreover, under the experimentally accessible modulation frequencies, the RC time constant associated with the graphene channel and measurement circuit is not expected to dominate the temporal response. In other words, the IEA structure enhances the optical driving field rather than altering the intrinsic carrier or thermal relaxation pathways of the detector.

The noise power spectral density of the device was further characterized by connecting the device to a low-frequency noise testing system (Figure 3c), showing that thermal noise dominates within the effective measurement range. From the measured noise spectrum and responsivity, the noise equivalent power (NEP) was determined to be 27 nW/Hz^0.5^ (Section 2). This relatively small NEP can be understood from several aspects of the device design. First, the compact PPAC architecture avoids the incorporation of external antennas or metamaterial absorbers, which helps maintain a small device footprint and limits parasitic contributions associated with larger coupling structures. Second, the PTE detection mechanism operates without an external bias, thereby suppressing additional noise sources such as bias-induced Johnson heating and excess electrical noise. Together with the cavity-enhanced absorption provided by the IEA structure, these features enable an improved signal-to-noise ratio. To evaluate operational stability, the detector was subjected to repeated on/off THz illumination cycles over 10 min. The corresponding switching response remains highly reproducible throughout the measurement (Figure 3d), with only minor peak-to-peak fluctuations attributable to small variations in the output power of the THz source, indicating excellent operational stability.

Compared with previously reported room-temperature graphene THz detectors (Figure 3e,f, and Table S1, Supporting Information), the present device exhibits moderate responsivity and NEP. These results indicate that the current architecture should be viewed primarily as a proof of concept for FP cavity-enhanced intrinsic plasmonic absorption rather than a fully optimized detector platform. The responsivity demonstrated here is also unlikely to represent the upper limit of the IEA-enhanced PPAC design. Previous studies have shown that shortening the channel length of PPAC devices can further improve the responsivity, refs. [26,29,44], indicating substantial room for optimization. In addition, our detector is based on large-area monolayer graphene grown by chemical vapor deposition (CVD), which offers better scalability and array compatibility than many previously reported THz detectors based on mechanically exfoliated graphene [26,45,46]. We therefore emphasize that the main advance of this work is not the simultaneous optimization of all detector figures of merit, but the demonstration that vertical cavity mode control can directly enhance intrinsic graphene plasmon absorption. The moderate absolute responsivity and NEP leave considerable room for further improvement through optimization of graphene quality, carrier asymmetry, thermal design, channel geometry, and impedance matching. Nevertheless, the nearly 30-fold enhancement relative to the non-cavity reference establishes vertical electromagnetic resonance mode control as an effective and broadly compatible strategy for improving graphene plasmonic THz detectors.

**Figure 4:**
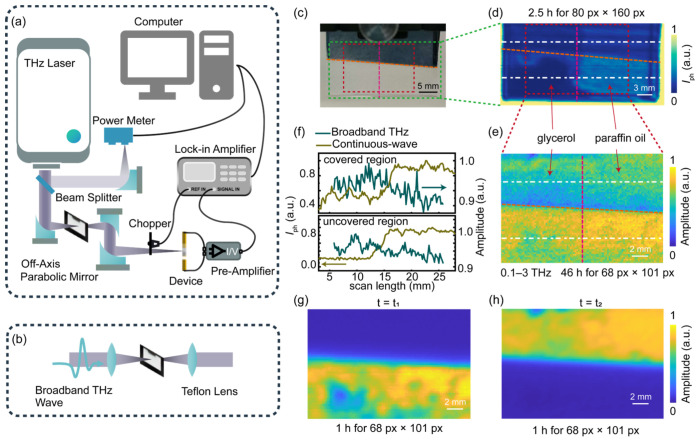
THz imaging of concealed polar and nonpolar liquids using the IEA-enhanced graphene detector. (**a**) Schematic of the continuous-wave photocurrent dual-focus scanning imaging setup. (**b**) Schematic of the broadband THz-TDS imaging setup. (**c**) Photograph of the sample used for imaging: a liquid-loaded filter paper partially covered by a high-resistivity silicon wafer. The magenta dashed line in panels (**c**–**e**) marks the boundary between the glycerol region (left) and the paraffin oil region (right). (**d**) Continuous-wave THz image of the sample acquired with the IEA graphene detector. The white dashed line in panels (**d**,**e**) represents the line profiles of the extracted covered and uncovered areas in panel (**f**). (**e**) Frequency-domain image of the same sample reconstructed from broadband THz-TDS measurements. (**f**) Line profiles extracted along the white dashed lines in panels (**d**,**e**). The line profiles of the dual-focal scanning correspond to the left coordinate axis, and the line profiles of the TDS iamge correspond to the right coordinate axis, as indicated by the same-colored arrows. (**g**,**h**) Time-gated THz-TDS images of the sample reconstructed at delays *t*_1_ (**g**) and *t*_2_ (**h**), respectively.

The IEA-enhanced graphene detector exhibits robust performance for THz imaging applications, enabling nondestructive and noncontact imaging that is valuable for distinguishing materials with different compositions. As a proof of concept, the detector was used to image two concealed liquids with distinct chemical properties. Glycerol and paraffin oil, which are polar and nonpolar liquids, respectively, were selected because their different polarities lead to markedly different THz refractive indices [47,48]. To prepare the sample, a 55-μm thick filter paper was placed on a 1 mm thick quartz substrate with a size of 3 cm × 2 cm (Figure 4c). Approximately 100 μL of glycerol and paraffin oil were separately absorbed onto the left and right sides of the filter paper, respectively, and allowed to spread uniformly. The upper half of the sample was then covered with a high-resistivity silicon wafer (resistivity > 20,000 Ω·cm), with the boundary marked by the orange dashed line in Figure 4c. In the following discussion, the upper and lower halves of the sample are referred to as the covered and uncovered regions, respectively.

The sample was mounted on a motorized translation stage, and THz imaging was performed under 2.52 THz continuous-wave illumination using the dual-focus scanning configuration (Figure 4a). The scan step size was fixed at 200 μm, which defines the effective spatial resolution of the present imaging experiment. The detector photocurrent was amplified, demodulated by a lock-in amplifier to suppress noise, and recorded through a data-acquisition system (Section 2). The normalized imaging result shows that continuous-wave dual-focus THz scanning with the IEA-enhanced graphene detector clearly distinguishes the two liquids, including in the covered region (Figure 4d). This contrast arises from the markedly different THz dielectric responses of the two liquids. Glycerol, as a polar liquid, exhibits much stronger absorption and typically a higher refractive index in the THz range than nonpolar paraffin oil. As a result, the THz wave experiences greater attenuation in the glycerol region, leading to a lower transmitted signal and hence a smaller photocurrent at the detector. This difference produces clear image contrast between the two regions, as further evidenced by the line profiles taken across the covered and uncovered areas (Figure 4f).

For comparison, the same sample was also characterized using a transmission-mode THz time-domain spectroscopy (THz-TDS) imaging system (Figure 4b), where a full time-domain waveform was acquired at each pixel. THz-TDS imaging is a widely used technique for distinguishing materials with different chemical compositions. The recorded temporal signals (Figure S6, Supporting Information) were Fourier transformed to obtain spectra from 0.1 to 3 THz, and the spectral intensity was then integrated to generate a broadband TDS image. Compared with the continuous-wave result, the TDS image shows substantially weaker contrast between the two liquid regions under the present imaging conditions (Figure 4e). This difference is also evident in the corresponding line profiles extracted from the dashed lines in Figure 4d–f. After normalization, the image contrast obtained by continuous-wave scanning is 4.2 times larger than that of the broadband TDS image.

Imaging speed is another important metric for evaluating practical THz imaging capability. For an imaging area of 80 × 160 pixels with a step size of 200 μm, the continuous-wave method requires approximately 2.5 h, whereas the THz-TDS measurement under a similar spatial step but smaller scan area (68 × 101 pixels) requires about 46 h. This corresponds to a 34-fold improvement in imaging speed per unit area for the 2.52 THz continuous-wave scanning approach. The large time cost of THz-TDS mainly originates from the need to record a complete temporal waveform at every pixel in order to reconstruct the spectral response. In the present sample geometry, the silicon cover introduces additional interfaces and optical path differences, which further complicate TDS-based imaging. Specifically, the detected time-domain waveform contains temporally separated contributions associated with different propagation paths, including signals arriving from the front surface of the silicon cover and from the sample region beneath it. If the image is reconstructed by selecting a fixed temporal gate centered at a specific time delay corresponding to one of the two main transmission features, such as *t*_1_ or *t*_2_, only one portion of the time-resolved signal is sampled. Although this gating approach can reduce acquisition time, the resulting images at *t*_1_ and *t*_2_ show opposite contrast between the covered and uncovered regions, making it difficult to unambiguously identify the two liquids from a single time-gated image. More generally, when a sample contains regions with significantly different refractive indices or layer thicknesses, the local THz arrival time shifts from pixel to pixel. Under such conditions, single-delay imaging becomes unreliable, and multiple gated acquisitions or full waveform measurements are required, which further increases the measurement burden. By contrast, continuous-wave imaging is not affected by this temporal path-separation problem, because it directly probes the transmitted amplitude at a fixed frequency rather than reconstructing the signal from time-resolved waveforms. As long as the cover layer does not eliminate the transmission contrast of the concealed object, the continuous-wave scheme can rapidly distinguish materials with different THz dielectric responses. These results highlight the practical advantage of the IEA-enhanced graphene detector for rapid THz imaging of concealed objects: although the continuous-wave scheme sacrifices the spectral completeness of broadband THz-TDS, it provides substantially higher throughput and stronger application-specific contrast when fast material discrimination is required.

## 4. Conclusions

In conclusion, we demonstrate an interferometric enhancement of the absorption strategy for graphene plasmon polariton atomic cavity THz detectors by integrating a metallic back reflector beneath a silicon substrate of designed thickness to form an FP cavity. This cavity positions the standing-wave antinode at the graphene plasmonic layer, thereby redistributing the vertical standing-wave field and maximizing the local in-plane driving field responsible for intrinsic graphene plasmon excitation. As a result, the IEA-enhanced PPAC device exhibits a 30-fold increase in responsivity while preserving a response time below 130 μs.

Unlike previously reported FP cavity-assisted graphene detectors that rely on antennas or metamaterial resonators for light coupling, the present architecture enhances detection by directly controlling the excitation amplitude of intrinsic graphene plasmons through cavity-engineered vertical electromagnetic boundary conditions while leaving the intrinsic plasmon resonance frequency solely governed by the lateral graphene geometry. In this scheme, the plasmon resonance frequency remains set by the lateral geometry of the graphene nanostructure, whereas its excitation amplitude is governed by the cavity-defined out-of-plane field distribution. This separation of resonance frequency and excitation strength establishes a simple and broadly compatible route for enhancing intrinsic plasmon absorption without auxiliary metallic resonators.

The resulting detector enables rapid discrimination of concealed polar and nonpolar liquids at 2.52 THz, delivering more than 30-fold higher imaging throughput and over four-fold stronger contrast than broadband time-domain measurements. Beyond the present device performance, this work identifies vertical cavity mode control as an effective design principle for graphene plasmonic THz photodetectors and provides a scalable pathway toward room-temperature THz sensing and imaging based on large-area graphene platforms.

## Data Availability

The original contributions presented in this study are included in the article/Supplementary Materials. Further inquiries can be directed to the corresponding author.

## Supplementary Materials

The following supporting information can be downloaded at: https://www.mdpi.com/article/10.3390/nano16130794/s1, Figure S1: Matching of theoretical calculations and numerical simulation results; Figure S2: Simulated temperature distribution on the surface of the IEA device; Figure S3: Fabrication process flow of the device; Figure S4: Device’s resistance distribution; Figure S5: Simulated local field difference squared between electrodes at graphene channel ends; Figure S6: Time-domain signals of the glycerol film; Figure S7: Raman spectra of graphene within the device channel; Figure S8: Output power curve of the electronic source; Figure S9: Cross-sectional SEM image of the silicon substrate; Table S1: Comparison of representative graphene-based detectors [26,29,32,33,45,46,49,50,51,52,53,54,55].

## Abbreviations

2D, two-dimensional; THz, terahertz; PPAC, plasmon polariton atomic cavity; FP, Fabry–Pérot; IEA, interferometric enhancement of absorption; PTE, photothermoelectric; FDTD, finite-difference time-domain; PEC, perfect electric conductor; NEP, noise equivalent power; CVD, chemical vapor deposition; THz-TDS, terahertz time-domain spectroscopy; SEM, scanning electron microscopy.
