# Supplementary material for: Interference-Enhanced Absorption in Miniaturized Graphene Plasmonic Terahertz Detectors via Substrate-Defined Fabry−Pérot Cavities
Source: Nanomaterials (Basel). 2026 Jun 26;16(13):794. doi: 10.3390/nano16130794 (PMC13363448; doi:10.3390/nano16130794)
Supplement: Supplementary file 1 [file nanomaterials-16-00794-s001.zip › nanomaterials-4326190-supplementary.pdf]

Supporting Information for

Interference-Enhanced Absorption in Miniaturized Graphene Plasmonic Terahertz Detectors via Substrate-Defined Fabry–Pérot Cavities

Runli Li [1,2,†], Shaojing Liu [1,2,†], Ximiao Wang [1,2], Hongjia Zhu [1,2], Yongsheng Zhu [1,2], Shangdong Li [1,2], Huanjun Chen [1,2], and Shaozhi Deng [1,2]*

[1] State Key Laboratory of Optoelectronic Materials and Technologies, School of Electronics and Information Technology, Sun Yat-Sen University, Guangzhou 510275, China;

[2] Guangdong Province Key Laboratory of Display Material and Technology, School of Electronics and Information Technology, Sun Yat-Sen University, Guangzhou 510275, China

*Corresponding authors: chenhj8@mail.sysu.edu.cn.

[†]These authors contributed equally.

Number of pages: 13

Number of figures: 9

Number of tables:1

CONTENTS

Supplementary Figures S3

S1. Matching of theoretical calculations and numerical simulation results. S3

S2. Simulated temperature distribution on the surface of the IEA device for different substrate thicknesses. S4

S3. Fabrication process flow of the device. S5

S4. Devices resistance distribution. S6

S5. The square of the local field difference between the electrodes in contact with the two ends of the graphene channel under different terahertz wave polarization directions. S7

S6. Time-domain signals of the glycerol film with and without silicon cover. S8

S7. Raman spectra of graphene within the device channel and in the etched regions. S9

S8. Output power curve of the electronic source. S10

S9. Cross-sectional SEM image of the silicon substrate. S11

Supplementary Tables S12

S1. Comparison of representative graphene-based room-temperature THz detectors. S12

Supplementary Figures

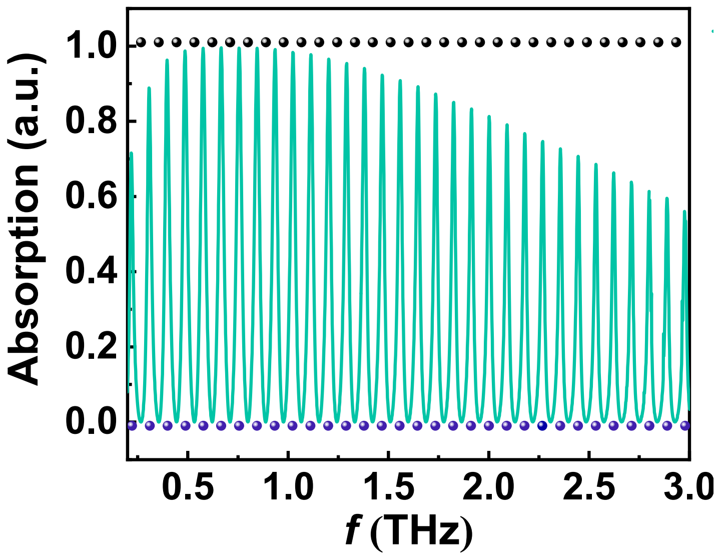

Figure S1. Matching of theoretical calculations and numerical simulation results. The solid green line in the figure represents the absorption efficiency of the graphene disk with IEA structure in the terahertz band under FDTD simulation, the purple dots represent the peak positions of coherent absorption enhancement calculated by Equation 7, and the black dots are the positions where half-wave loss is removed, i.e., the positions of coherent destructive phases. It can be seen that the two calculated results match well with the FDTD simulation results, and the slight error can be attributed to the proximity selection of the simulation mesh.

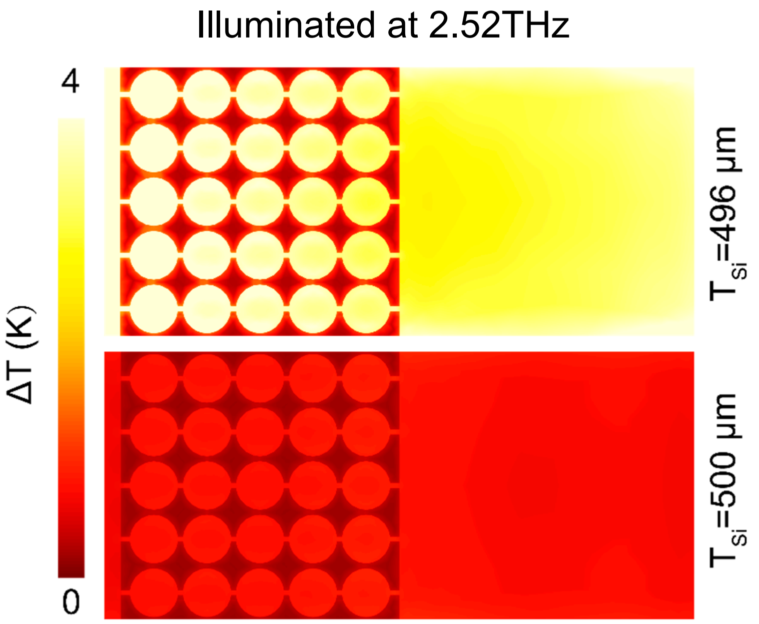

Figure S2. Simulated temperature distribution on the surface of the IEA device for different substrate thicknesses. ΔT represents the difference between the device temperature and room temperature (300 K).

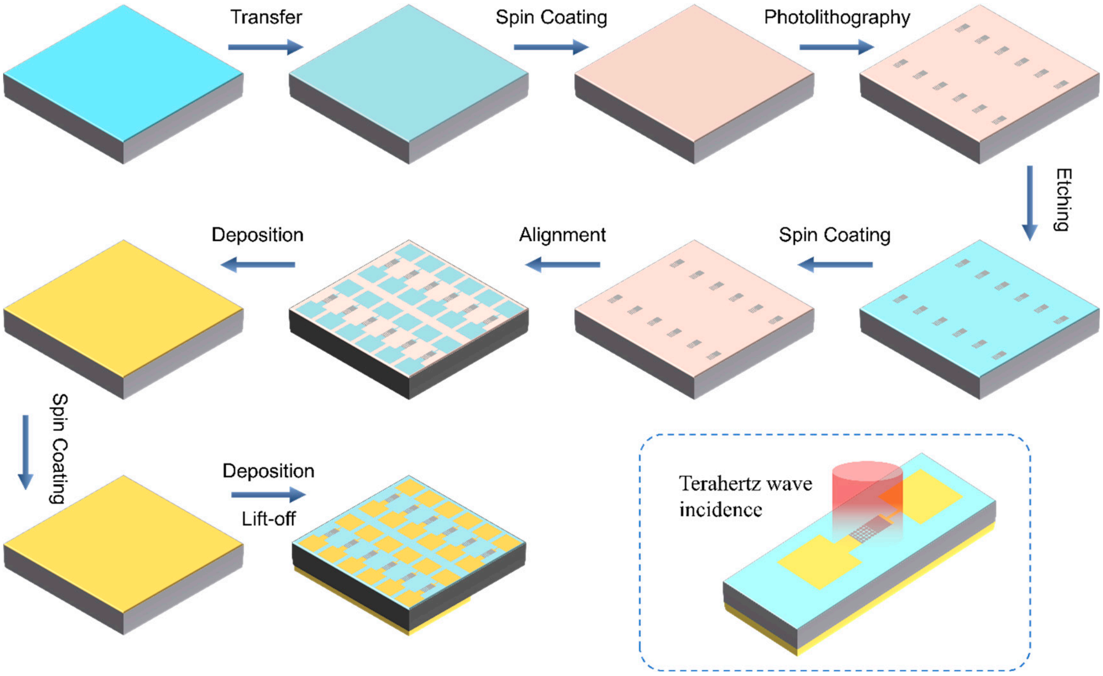

Figure S3. Fabrication process flow of the device.

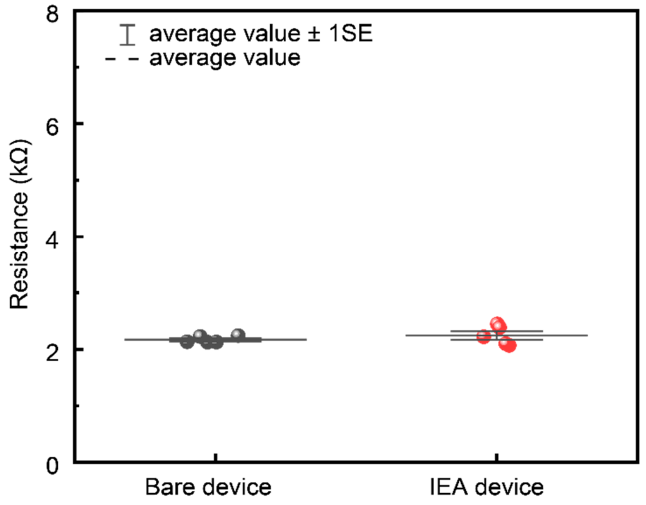

Figure S4. Statistics on the devices resistance distributions. The coefficient of variation of the device resistance remains below 25%.

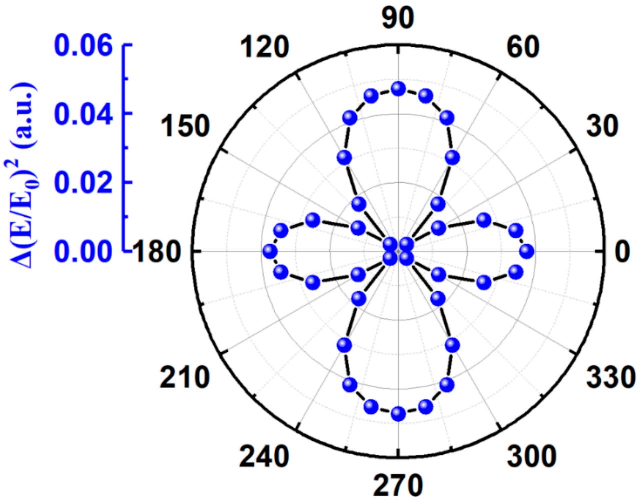

Figure S5. The square of the local field difference between the electrodes in contact with the two ends of the graphene channel under different THz wave polarization excitations.

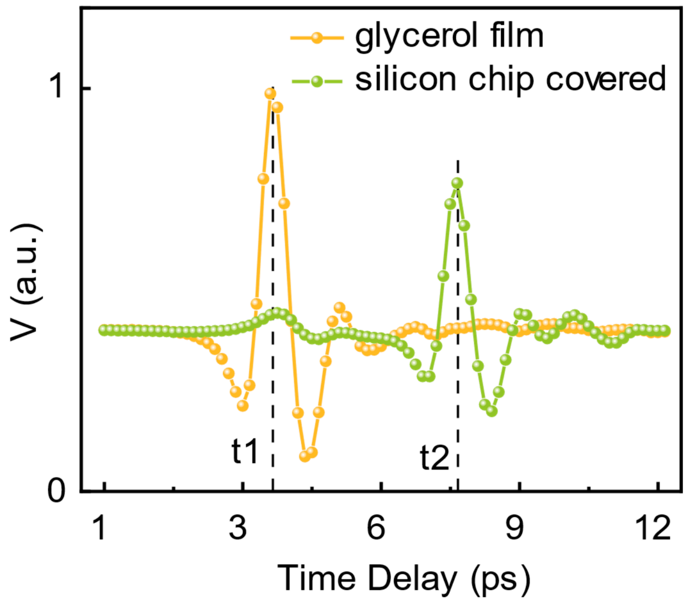

Figure S6. Time-domain signals of the glycerol film with (light green) and without silicon cover (orange).

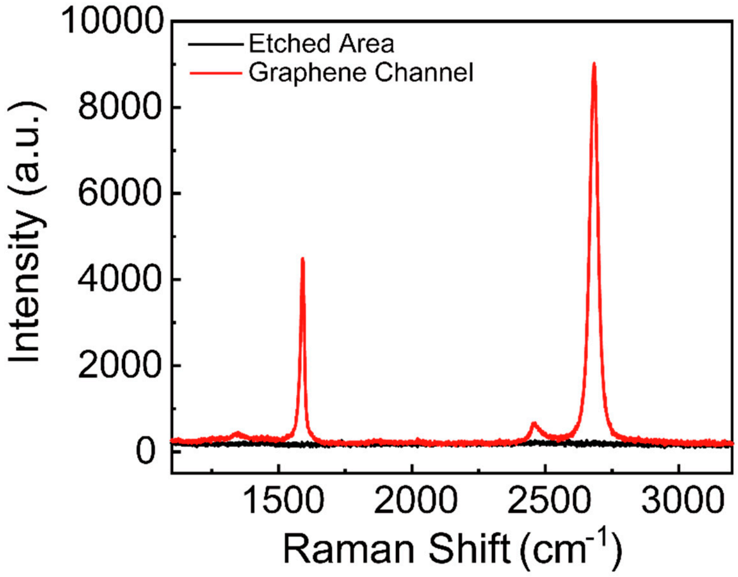

Figure S7. Raman spectra of graphene within the device channel and in the etched regions.

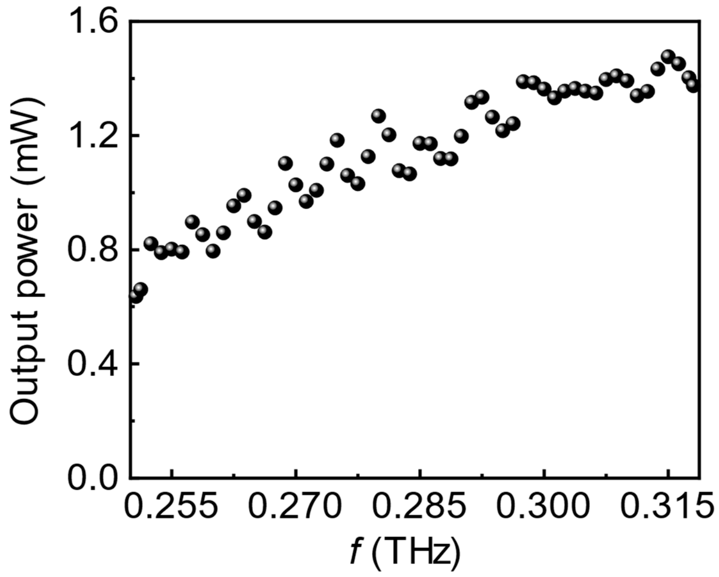

Figure S8. Output power of the THz electronic source at different frequency.

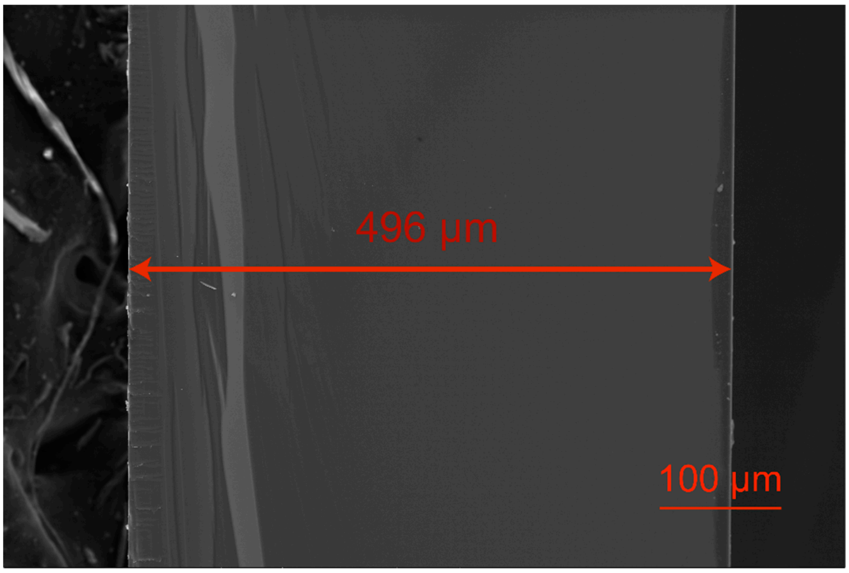

Figure S9. Cross-sectional SEM image of the silicon substrate.

Supplementary Tables

Table S1. Comparison of representative graphene-based room-temperature THz detectors

Device architecture	Responsivity	Frequency (THz)	Response time (s)	Channel area (μm^2)	NEP ($nW/Hz^{0.5}$)	Reference
FP cavity	0.0033 V/W	2.52, 0.25–0.32	10^{-4}	51,200	27	This work
Flexible substrate	0.0337 V/W	0.25–923	10^{-1}	1,000	8.6	1
Metasurface	0.0085 V/W	2	-	31,365	-	2
Exfoliated material	0.5 V/W	1.63–3.11	-	7.6	-	3
Exfoliated material	700 V/W	2.52	10^{-10}	9	0.02	4
PPAC	2.48 V/W	0.22–4.24	10^{-8}	51,925	2.8	5
Metasurface	0.14 V/W	0.6–0.31	-	460	100	6
Metasurface	400 V/W	0.04, 0.12, 0.3, 0.6	10^{-5}	750,000	0.7	7
Metasurface FP cavity	0.36 V/W	2.52	10^{-4}	30,000	13.1	8
Metasurface FP cavity	1.26 V/W	1.6	-	4,500	5.2	9
Metasurface	14.12 V/W	0.08–0.75	10^{-6}	42,4000	0.08	10
Antenna Metasurface	3.16 V/W	2.52, 3.11	10^{-5}	30,000	9.3	11
Metasurface	2 mA/W	2, 2.7	-	1,200,000	-	12
Antenna Metasurface	34 μA/W	2	10^{-3}	1,200,000	150	13

Reference

(1) Liu, S.; Wang, X.; Xu, N.; Li, R.; Ou, H.; Li, S.; Zhu, Y.; Ke, Y.; Zhan, R.; Chen, H.; et al. A Flexible and Wearable Photodetector Enabling Ultra-Broadband Imaging from Ultraviolet to Millimeter-Wave Regimes. *Advanced Science* **2024**, *11* (26), e2401631. DOI: 10.1002/advs.202401631.
(2) Xiao, L.; Degl'Innocenti, R.; Wang, Z. Key Factors in Achieving High Responsivity for Graphene‐Based Terahertz Detection. *Advanced Photonics Research* **2024**, *5* (8). DOI: 10.1002/adpr.202300272.
(3) Muraviev, A. V.; Rumyantsev, S. L.; Liu, G.; Balandin, A. A.; Knap, W.; Shur, M. S. Plasmonic and bolometric terahertz detection by graphene field-effect transistor. *Applied Physics Letters* **2013**, *103* (18). DOI: 10.1063/1.4826139.
(4) Cai, X.; Sushkov, A. B.; Suess, R. J.; Jadidi, M. M.; Jenkins, G. S.; Nyakiti, L. O.; Myers-Ward, R. L.; Li, S.; Yan, J.; Gaskill, D. K.; et al. Sensitive room-temperature

terahertz detection via the photothermoelectric effect in graphene. *Nature Nanotechnology* **2014**, *9* (10), 814-819. DOI: 10.1038/nnano.2014.182
(5) Chen, H.; Wang, X.; Liu, S.; Cao, Z.; Li, J.; Zhu, H.; Li, S.; Xu, N.; Deng, S. Monolithic Multiparameter Terahertz Nano/Microdetector Based on Plasmon Polariton Atomic Cavity. *Advanced Materials* **2025**, *37* (11), e2410946. DOI: 10.1002/adma.202410946.
(6) Mönch, E.; Hubmann, S.; Yahniuk, I.; Schweiss, S.; Bel'kov, V. V.; Golub, L. E.; Huber, R.; Eroms, J.; Watanabe, K.; Taniguchi, T.; et al. Nonlinear intensity dependence of ratchet currents induced by terahertz laser radiation in bilayer graphene with asymmetric periodic grating gates. *Journal of Applied Physics* **2023**, *134* (12). DOI: 10.1063/5.0165248.
(7) Yu, A.; Yang, Z.; Cai, M.; Zhang, H.; Tian, Z.; Guo, X.; Wang, L.; Balakin, A. V.; Shkurinov, A. P.; Zhu, Y. Graphene plasmons-enhanced terahertz response assisted by metallic gratings. *Nanophotonics* **2022**, *11* (21), 4737-4745. DOI: 10.1515/nanoph-2022-0455
(8) Chen, M.; Wang, Y.; Zhao, Z. Localized Electromagnetic Resonance Enabled THz Photothermoelectric Detection in Graphene. *Frontiers in Physics* **2020**, *8*. DOI: 10.3389/fphy.2020.00216.
(9) Alihosseini, F.; Zandi, H. Enhanced Spectrally Selective Terahertz Detection in a Photo-Thermoelectric Graphene Detector With Dual Nano-Grating Gates. *IEEE Journal of Quantum Electronics* **2024**, *60* (6), 1-8. DOI: 10.1109/jqe.2024.3462953.
(10) Meng, S.; Wang, L.; Zhang, H.; Yu, A.; Pan, X.; Zhang, Q.; Guo, X.; Balakin, A. V.; Shkurinov, A. P.; Zhu, Y. Toward non-degraded broadband room temperature terahertz detection by graphene plasmon-enhanced photo-thermoelectric effect. *Chinese Journal of Physics* **2024**, *89*, 1571-1577. DOI: 10.1016/j.cjph.2024.04.006.
(11) Chen, M.; Wang, Y.; Zhao, Z. Monolithic Metamaterial-Integrated Graphene Terahertz Photodetector with Wavelength and Polarization Selectivity. *ACS Nano* **2022**, *16* (10), 17263-17273. DOI: 10.1021/acsnano.2c07968.
(12) Degl'Innocenti, R.; Xiao, L.; Kindness, S. J.; Kamboj, V. S.; Wei, B.; Braeuninger-Weimer, P.; Nakanishi, K.; Aria, A. I.; Hofmann, S.; Beere, H. E.; et al. Bolometric detection of terahertz quantum cascade laser radiation with graphene-plasmonic antenna arrays. *Journal of Physics D: Applied Physics* **2017**, *50* (17). DOI: 10.1088/1361-6463/aa64bf.
(13) Degl'Innocenti, R.; Xiao, L.; Jessop, D. S.; Kindness, S. J.; Ren, Y.; Lin, H.; Zeitler, J. A.; Alexander-Webber, J. A.; Joyce, H. J.; Braeuninger-Weimer, P.; et al. Fast Room-Temperature Detection of Terahertz Quantum Cascade Lasers with Graphene-Loaded Bow-Tie Plasmonic Antenna Arrays. *ACS Photonics* **2016**, *3* (10), 1747-1753. DOI: 10.1021/acsphotonics.6b00405.
